# The CB_2_ Receptor as a Novel Therapeutic Target for Epilepsy Treatment

**DOI:** 10.3390/ijms22168961

**Published:** 2021-08-20

**Authors:** Xiaoyu Ji, Yang Zeng, Jie Wu

**Affiliations:** 1 Brain Function and Disease Laboratory, Shantou University Medical College, Xin-Ling Road #22, Shantou 515041, China; xyji@stu.edu.cn; 2 Medical Education Assessment and Research Center, Shantou University Medical College, Xin-Ling Road #22, Shantou 515041, China; yz@stu.edu.cn

**Keywords:** cannabinoid receptor 2, epilepsy, cAMP, M-current, anti-inflammatory

## Abstract

Epilepsy is characterized by repeated spontaneous bursts of neuronal hyperactivity and high synchronization in the central nervous system. It seriously affects the quality of life of epileptic patients, and nearly 30% of individuals are refractory to treatment of antiseizure drugs. Therefore, there is an urgent need to develop new drugs to manage and control refractory epilepsy. Cannabinoid ligands, including selective cannabinoid receptor subtype (CB_1_ or CB_2_ receptor) ligands and non-selective cannabinoid (synthetic and endogenous) ligands, may serve as novel candidates for this need. Cannabinoid appears to regulate seizure activity in the brain through the activation of CB_1_ and CB_2_ cannabinoid receptors (CB_1_R and CB_2_R). An abundant series of cannabinoid analogues have been tested in various animal models, including the rat pilocarpine model of acquired epilepsy, a pentylenetetrazol model of myoclonic seizures in mice, and a penicillin-induced model of epileptiform activity in the rats. The accumulating lines of evidence show that cannabinoid ligands exhibit significant benefits to control seizure activity in different epileptic models. In this review, we summarize the relationship between brain CB_2_ receptors and seizures and emphasize the potential mechanisms of their therapeutic effects involving the influences of neurons, astrocytes, and microglia cells. The unique features of CB_2_Rs, such as lower expression levels under physiological conditions and high inducibility under epileptic conditions, make it an important target for future research on drug-resistant epilepsy.

## 1. Introduction

Epilepsy is the third most common chronic neurological disorder that affects over 70 million people worldwide [1]. Occurrence of epileptic seizure in different brain areas may lead to loss of consciousness, motor or sensory disorders, and emotional or cognitive dysfunction [2]. Despite much progress in medical treatment using antiseizure drugs to control epileptic seizures, it still remains at 30% of patients that fail to be controlled by or respond to antiseizure drugs [3]. Thus, there is an urgent need to develop new medicines to control refractory epilepsy.

More understanding of underlying mechanisms in epileptogenesis has identified cellular and molecular targets for new therapies, for example, anti-inflammatory drugs that can overcome the limitations of current drugs and provide symptomatic control of epileptic seizures [4]. Accumulating data have demonstrated that cannabinoid systems, including endocannabinoids, anandamide, and 2-arachidonoyl glycerol, and their targets, the cannabinoid receptor subtype 1 (CB_1_R) and subtype 2 (CB_2_R), appear to regulate seizure activity [5,6,7,8,9,10,11,12,13]. The rationale for the antiseizure effects of the cannabinoid system is that the CB_1_Rs (possibly also CB_2_Rs) are linked to an inhibitory G protein (G_i/o_) signaling, which reduces neuronal excitability and/or neural synchronization. For example, the activation of brain CB_1_R modulates A-type K^+^ channels and N- and P/Q-type voltage-gated Ca^2+^ currents and stabilizes the membrane potentials [14,15], and it modulates presynaptic neurotransmitter release [16,17,18]. Furthermore, cannabidiol has been shown to not only reduce the frequency of seizures in animal models of epilepsy but also greatly decrease the frequency of drop seizures among children and adults with Lennox–Gastaut syndrome [19]. Based on these concepts, numerous cannabinoid analogues have been examined in a variety of animal models [5,8,11,20,21,22,23]. However, although cannabinoid ligands and CB_1_R agonists possess some antiseizure effects, non-specific modulations of cannabinoid systems will limit their therapeutic use for treatment of human epilepsy because of their severe adverse effects, for example, THC (50 mg or more) has been shown to lead to anxiety, psychosis, heart attack, and irregular heart rhythm. Regularly taking large amounts of cannabis over a long period of time might cause a cannabinoid hyperemesis syndrome. Therefore, significant attention is currently being directed toward the possibility of developing medicines from compounds that can selectively activate CB_2_Rs and have important potential therapeutic applications at doses that induce little or no CB_1_R-mediated effects.

In this review, we summarize the current state of knowledge on CB_2_R expression and function, which could serve as an important tool for intervention and control of seizure activity by modulating neuronal excitability and neuroinflammation.

## 2. CB_2_R Expression and Inducible Feature

Cannabinoid receptor type 2 (CB_2_R) is a plasma membrane G protein-coupled receptor that was characterized from spleen by Munro [24]. The expression and function of CB_2_ in the brain have been debated due to early studies implying that CB_2_Rs were missing in the central nervous system (CNS), since CB_2_R mRNA expression was not measured in rat brains by using in situ hybridization [24]. In accordance with this result, Northern blot analysis also failed to detect CB_2_R mRNA in rat, mouse, and human brains [25,26,27]. RT-PCR experiments demonstrated abundant CB_2_R expression among peripheral immune tissues, such as on spleen T cells and on macrophages, but barely measurable levels in rodent brains [25,26,28,29]. Little is revealed about CB_2_Rs receptor expression in microglia, astrocytes, and astrocytoma, and the activation of these receptors affecting cellular function and activity [28]. Based on the above research, CB_2_Rs have been classically considered as peripheral cannabinoid receptors [24,30,31]. Recently, this concept of CB_2_ deficiency within the brain has been challenged along with the identification of CB_2_Rs widespread in the CNS, though they are expressed at lower densities than CB_1_. Emerging evidence shows that significant CB_2_R mRNA can be detected by ISH in cultured granule cells among the granule layer and Purkinje cell layer of the mouse cerebellum [29], in the mouse retina [32], and in the globus pallidus of non-human primates [30]. RT-PCR analysis has also been applied to distinguish CB_2_ mRNA expression in multiple brain regions, including the retina [32], cortex [30,31,33,34], striatum [26,34], hippocampus [30], amygdala [33,34], brainstem [35], and cerebellum [36]. Furthermore, two CB_2_ isoforms, CB_2A_ and CB_2B_, have been characterized in the rodent and human brain [31] along with a new CB_2_ transcript that has been found in mouse and monkey B lymphocytes [37]. This suggests the possibility that CB_2_R expression not only exists in peripheral tissues, but also in the brain. It has been reported that CB_2_Rs modulate a variety of important processes in dopamine (DA)-related behaviors [38], including food intake [39,40,41,42], anxiety [33,43], depression [44], and schizophrenia-like behavior [34,45]. Recent evidences emerging from several laboratories, including ours, have indicated that brain CB_2_Rs play a pivotal role in the reduction of cocaine, alcohol, and nicotine addiction [46,47,48]. Collectively, these lines of evidence strongly suggest an important role of CB_2_R in the mesocorticolimbic system, as well as in various brain functions involving psychiatric, cognitive, and neurobiological activity. Compared to CB_1_Rs, central CB_2_Rs display the following unique features: (1) low expression grades, suggesting that they may not modulate neural functions under physiological conditions, (2) high pathological expression, meaning that under some pathological conditions (for example, addiction, stroke, stress, schizophrenia, inflammation, anxiety), CB_2_R expression increased in the brain [49], suggesting that the change of CB_2_R expression/function is closely related to various mental and neurological diseases, and (3) post-synaptic localization, where CB_2_R is mainly expressed in the neuronal somatodendritic area [50], whereas CB_1_Rs are chiefly expressed on neuronal terminals, especially on GABAergic terminals (presynaptic), which leads to some opposing effects after activation by these two receptor subtypes [51]. In consideration of these features, CB_2_R is likely an important target for neuroprotection [52], and targeting CB_2_Rs likely provide a novel therapeutic strategy for treating neuropsychiatric and neurological diseases without typical CB_1_-mediated side-effects. However, to fulfill this possibility, an understanding of the functional effects of CB_2_Rs in the brain is required. Unfortunately, the function of CB_2_Rs in the CNS has not been well-established, and studies of the functional effects of CB_2_Rs in neurons have ignited debate and controversy. A consensus has yet to emerge regarding the expression and function of CB_2_Rs in midbrain ventral tegmental area (VTA) neurons, which are the source of mesocorticolimbic dopamine (DA) signaling. In our recent study, we found that functional CB_2_Rs are expressed in VTA DA neurons, and the activation of these CB_2_Rs reduced the excitability of DA neurons through both intrinsic and synaptic mechanisms [53].

## 3. Drug Resistance in Epilepsy

The occurrence of epilepsy is usually related to a disorder of excitatory and/or inhibitory neurotransmitters, such as upregulation of glutamate and acetylcholine, and downregulation of GABA and serotonin. According to this concept, about 40 anti-seizure drugs are used for symptomatic treatment of epilepsy [54]. However, over one third of epileptic patients are resistant to multiple antiseizure drug therapies. Drug-resistant epilepsy can be defined as a failure of multiple efficient treatments of tolerated, chosen, and appropriately used anti-epileptic drug guidelines [55]. Based on the inducement and hypothetical mechanisms of drug-resistant epilepsy, recent speculations of mechanisms of drug resistance include: (1) Drug target variation: drug targets evoke the alteration of neurotransmitter receptors, voltage-dependent ion channels, and transporters participating in the metabolic pathway involved in the metabolism of neurotransmitters [56]. (2) Genetic mechanisms: The epigenome is a dynamic process, and endogenous mutations in receptor genes may be considered to cause the occurrence of drug-resistant epilepsy [57]. The roles in microbiome are also of great interest in epileptic disorders [58], and earlier research demonstrated that the effects of ketogenic diets for treatment of seizures may be involved in microbial processes [59]. (3) The drug target is missed because most of the anti-epileptic drugs focus on the neuronal inhibition and excitation but do not pay attention to the real pathogenesis caused by encephalitis or cancer. In view of patients with drug-resistant epilepsy, the alternative treatment methods mainly include: (1) surgical treatment via removal of epileptic foci [60], where with the development of epileptic area localization and imaging technology, as well as the in-depth research on the resistance mechanism of epilepsy, comprehensive treatment based on surgery will continue to be improved and promoted, (2) transcranial magnetic stimulation, where the combination of transcranial magnetic stimulation (TMS) and motor cortical EEG enables biomarkers to provide cortical stimulation and suppression measures that are particularly relevant to epilepsy [61], (3) an embedded stimulating electrode, applied to interfere with the synchronization process of abnormal nerve cells’ discharge [62] or for provocation of the electrical current to the vagus nerve [63], and (4) ketogenic diets, which imitate the fasting state by taking in fat as the chief fuel source therapy [64].

## 4. Cannabinoid System as a Potential Therapeutic Target for Treating Epilepsy

### 4.1. Endocannabinoid System

The endocannabinoid system (ECS) is involved in regulation of excitatory and inhibitory synaptic transmission in the brain [65,66], and consists of two G protein-coupled receptors, CB_1_R and CB_2_R, with two known endogenous cannabinoid ligands, namely 2-arachidonoylglycerol (2-AG) and *N*-arachidonoylethanolamide (NAN), respectively [10,11]. In the past few years, scientists have drawn attention to using a treatment focused on ECS [67,68]. There is already a comprehensive review indicating the roles of ECS dysfunction-induced neuroinflammation in epilepsy [69]. Anticonvulsant-like effects of cannabinoid receptor agonists are dependent on CB_1_R. CB_1_ agonists could increase ATP-sensitive K^+^ channel (K_ATP_) activation by decreasing mitochondrial ATP levels. CB_1_R-mediated regulation in neuronal excitability can exert antiseizure effects [70]. Microglia are evoked by pathogens, products of damaged/inflammatory neurons, and destruction of the blood–brain barrier, as well as diverse chemical menace signals. However, there is also a contradiction about suppressing epilepsy by adjusting the activity of CB_1_Rs to affect excitability. Owing to the extensive distribution and high level of CB_1_ in the CNS under physiological conditions, a risk of side-effects develops when CB_1_Rs are activated and targeted.

### 4.2. Cannabinoids’ Effects on Epilepsy

Tetrahydrocannabinol (THC) and cannabidiol (CBD) have been the most researched at present, especially in psychoactive pharmacology. THC and CBD have many similarities in structure, and their structure–activity relationship has an impact on mental activity. It has been reported that both have certain anticonvulsant effects. THC mainly activates the GPR55 receptor, and partially activates the CB_1_Rs and CB_2_Rs. However, THC can cause a series of psychological effects, such as coordination problems, slower reaction times, memory loss, anxiety, and addiction. CBD can reduce these side-effects caused by THC. Studies have reported that CBD treats the frequency of spontaneous seizures in DS mice mainly by improving the excitability of hippocampal interneurons, and the excitability of vertebral neurons in the dentate gyrus to strong depolarization stimulation is also reduced [67]. The pharmacological activity of CBD is mainly focused on blocking the effect of GPR55 receptors to inhibit the effect of neurotransmission and significantly reduce the α-amino-3-hydroxy-5-methyl-4-isoxazole propionic acid receptor (AMPAR)-mediated amplitude and frequency of induced excitatory postsynaptic currents (EPSCs) and miniature EPSCs (mEPSCs) [71]. CBD is also used clinically to treat epileptic seizures caused by Lennox-Gastaut syndrome [19]. A proportion of patients allocated to CBD treatment in these trials were receiving clobazam (CLB). Bialer and Perucca showed an antiseizure effect of CBD independent of an interaction with CLB. A greater antiseizure effect and greater adverse effects were observed when CBD was combined with CLB [72]. However, the therapeutic effect of CBD on other types of epilepsy and the complete mechanism of action remains unclear, but it may involve a non-CLB mechanism. The peripheral side-effect of CBD is mainly diarrhea. There is only indirect proof implying that CBD could modulate endocannabinoid signaling, but no promising data indicating a direct binding or interaction between CBD and CB receptors. CBD can also partially activate CB_2_Rs [73]. Negative allosteric modulation of CB_2_R activity by CBD might interpret its action for antiseizure and other neural disorders, which provides us with novel insights to develop its medical application [74]. Regarding CB_2_R, CBD-DMH, a modification on different pharmacophoric sties, was considered to promote a conformational change in CB_2_R, which favors G protein-dependent signaling rather than β-arrestin-dependent signaling [75]. However, there are very few modulators of CB_1_R and CB_2_R reported in the literature.

### 4.3. CB_2_R Effects on Preclinical Epilepsy

Since CB_2_Rs exhibit low expression levels in the brain under normal conditions, but are highly inducible during various disease states (including epilepsy), they appear to be an important substrate for neuroprotection [76]. Targeting CB_2_Rs will likely offer a novel therapeutic strategy for treating epileptic seizures without the typical CB_1_R-mediated side-effects [77,78]. Emerging evidence has indicated that CB_2_Rs are involved in epileptic activity in animal models. In an acute pentylenetetrazol (PTZ) rat seizure model, pretreatment with palmitoyl ethanolamide (PEA) increased the latency of seizure initiation and reduced the duration of seizures, and this antiseizure effect was attenuated by the CB_2_R antagonist (AM630), suggesting that CB_2_Rs mediate PEA’s effect [79]. In developing rats, Huizenga et al. examined the antiseizure effects of a variety of cannabinoid ligands, and found that either combined CB_1_R/CB_2_R or selective CB_1_R agonists exhibited antiseizure effects in either chemo-convulsing methyl-6,7-dimethoxy-4-ethyl-beta-carboline-3-carboxylate or PTZ seizure models of 10-day postnatal rats [80]. Although the CB_2_R selective agonist HU-308 did not show an antiseizure effect, the CB_2_R selective antagonist AM630 did increase seizure severity [80]. In addition, a recent report showed that CB_1_R knockout (KO) mice did not have an epilepsy phenotype, but co-KO of CB_1_R and CB_2_R caused animal epilepsy [81], suggesting that CB_2_R plays a role in stabilizing the neuronal system. Recent studies have also explored the effects of modulating CB_2_R activity on seizure susceptibility (Table 1). The activation of CB_2_Rs decreases excitatory synaptic transmission in the CNS. The new roles for CB_2_R have been identified in inducing hippocampal pyramidal cell hyperpolarization and inhibiting epileptic seizures [12]. The CB_2_Rs expressed on hippocampal CA3 neurons also play a critical role in reduced neuronal excitation and oscillations [82]. WIN 55212-2, a non-selective CB receptor agonist, shows striking antiseizure effects in a rat epileptic model [83], and CB_1_R and CB_2_R double-knockout mice show spontaneous or manual-evoked seizures [81]. CB_2_R knockout mice including both heterozygous and homozygous exhibit enhanced epileptic susceptibility, and a reduction in CB_2_R activity is associated with increased susceptibility [84], suggesting that absent CB_2_Rs can increase seizure susceptibility. The administration of caryophyllene, a CB_2_R agonist, was found to improve seizure activity in a mouse model [85]. Collectively, these lines of evidence support the idea that the activation of CB_2_Rs exhibits the antiseizure role. On the other hand, some studies reported different responses by CB_2_R agonists. For instance, HU-308 [80] and JHW133 [84] show no significant effect on mice seizure occurrence. Moreover, CB_2_R agonist AM1241 increases seizure intensity in a PTZ model [86]. Additionally, CB_2_R antagonists AM630 and SR144528 can increase seizure susceptibility [80]. Therefore, it is likely that the altered CB_2_R activity can affect seizure susceptibility, although the underlying mechanisms are still unclear. The different effects of CB_2_R-mediated modulations may come from the differences in using different types of CB_2_R ligands, and be based on different experimental designs, including species, epileptic model types, and dosage. We recently found that the commercially available CB_2_R agonists showed different effects on pancreatic acinar cell Ca^2+^ oscillations [87]. Nevertheless, numerous lines of evidence have demonstrated that CB_2_R agonists ameliorate a variety of epileptic seizures, suggesting that CB_2_R is a potential therapeutic target for treating epilepsy.

### 4.4. CB_2_R Mediated Anti-Epileptic Effects through a Reduction of Neural Excitability and Synchronization

As a crucial neuro-modulatory system of the brain, the midbrain dopaminergic system plays an important role in neuronal excitability. Temporal lobe epilepsy, involving pathological erethism of the hippocampus, is associated with VTA dopamine neuron activation [88]. The abnormally high synchronous activity of neuronal firing will cause phased impulse stimulation, causing more dopamine neurons to produce impulse firing, so that the dopaminergic system is in a super-reactive state during seizures’ occurrence [89]. This suggests that the dopaminergic system is vital in epileptic brains, and the evidence for a relationship between epilepsy and the dopaminergic system was previously described by Rezaei [2]. Pilocarpine-induced epileptic rats exhibit a significant enhancement in activity of dopaminergic neurons [88]. In the PTZ kindling model of epilepsy in mice, dopamine neurons within the VTA display hyperactivity when compared to saline-injected controls [90]. Anti-seizure treatments (antiseizure drugs or brain stimulation) are applied to downregulate the neuronal excitability for controlling epileptic seizures. 

Stimulation of G_s_-linked G protein-coupled receptors (GPCRs) triggers adenylate cyclase (AC) to produce cAMP from ATP. Activation of this signal pathway causes calcium influx through plasma membrane channels in a calmodulin-dependent manner [91]. Recent studies using transcriptomic analysis show that the transcription of a set of genes related to cAMP signaling is changed in patients suffering from drug-resistant temporal lobe epilepsy [92,93]. When seizures occur, levels of cAMP increase in the brain. The appearance of cAMP signaling can be divided into temporary influence on neuronal excitability, including ion channel or receptor phosphorylation [75], and long-term effects on epileptogenesis, such as effects regulated by cAMP response factor binding protein, CREB [94]. By coupling to G_i_ proteins, the activation of CB_2_Rs causes inhibition of AC activity and downregulation of cAMP release [95]. 5-HA_1A_-CB_2_ heteroreceptors were characterized in cortical primary cultures of neurons, and 5-HT_1A_R-CB_2_ heteroreceptor complex expression and functionality are significantly enhanced in the brain after cerebral ischemia, especially during the neonatal term, also suggesting that this heteromer is associated with NHIBD pathophysiology [96]. Therefore, CB_2_R activation may suppress the occurrence of epilepsy via decreasing excitability of the CNS by reducing the level of cAMP.

Most K^+^ channels are controlled by various physiological mediators, such as transmembrane voltage, and intracellular Ca^2+^ and G proteins. The roles of K^+^ in membrane physiology have been extensively investigated in rodent models, and the basic electrophysiological properties and bursting patterns of primate central neurons are generally similar to those reported for the rodent [97]. K^+^ channels are very important in regulating the intrinsic excitability of neurons, and they are the main contributor to neuronal membrane repolarization [98]. K^+^ channels are a promising target for the development of novel anti-epileptic drugs, and activation of the K^+^ channels can be used for restoring control on neuronal excitability in patients with epilepsy [99,100]. Kv7 (M-) channels are an unusual type of K^+^ channel which is different from those that repolarize an individual’s action potential. The M-channels are partially activated within the resting membrane potential of neurons, and further activated by membrane depolarization. The rate of M-channel open and close is slower than other types of K^+^ channels that contribute to the repolarization of action potentials. Activation of M-channels can inhibit highly synchronized neuronal firing that may reduce hyper-excitation and seizure activity [101]. The K^+^ channel opener retigabine is a compelling and selective opener of M-type K^+^ channels and is approved for therapy of drug-resistant focal and focal-to-bilateral tonic-clonic seizures [102]. In VTA DA neurons, G protein-coupled receptor signals regulate the excitability of neurons through a few ion channels, such as G protein-gated internally rectified K^+^ channels (GIRKs) [50,51,52]. The CB_2_R agonist JWH133 has been confirmed to effectively modulate the excitability of neurons by regulating voltage-dependent M-type K^+^ channels [53].

### 4.5. Glia CB_2_R-Mediated Anti-Epileptic Effects via Inflammation and Excitability

More and more lines of evidence show that CB_2_Rs are relevant to both immune cell competence at the peripheral region [103] and brain cells in the CNS. Actually, neurons, microglia, and astrocytes cells express CB_2_Rs [32,104,105], which are capable of modulating central neural-immune function and impact the related diseases [106,107]. It is critical to accentuate that CB_2_R has inducible expression in several immune cells under activated neuroinflammatory conditions. It means that CB_2_R levels may be upregulated in the CNS and increased in inflamed brain parenchyma due to the invasion of peripheral immune cells (such as peripheral T cells) that express CB_2_Rs [107]. During activated processes, microglia increase the expression of an array of membrane surfaces of CB_2_Rs that may be essential in microglial production and/or degeneration within the brain. Both CB_1_ and CB_2_ receptors were expressed on microglia using an in vitro assay, including immunoglobulin superfamily receptors, cell component receptors, toll-related receptors, opioid receptors, and cannabinoid receptors [13]. A notable example is that neuropathic pain upregulates CB_2_Rs in microglia in rat spinal cord, while chronic inflammatory pain does not [108]. When there is an inflammation response in the body, microglia CB_2_R is rapidly upregulated and activated, which effectively inhibits the release of harmful factors, including TNF-α and free radicals [103].

In patients with epilepsy, medial temporal lobe sclerosis, cortical dysplasia, encephalitis, and glioma, the astrocytes show increased expression of CB_2_Rs, which may change in K^+^ currents during seizures. It may lead to overexcitation and changes in a series of enzymatic pathways, which means that astrocytes may change the M-type K^+^ current by adjusting the expression of CB_2_Rs to change the intensity of epilepsy. We believe that the role of the astrocyte/astrocytic CB_2_R–cAMP signaling pathway in controlling epileptogenesis is worthy of further exploration.

## 5. Conclusions and Perspective

**Figure 1:**
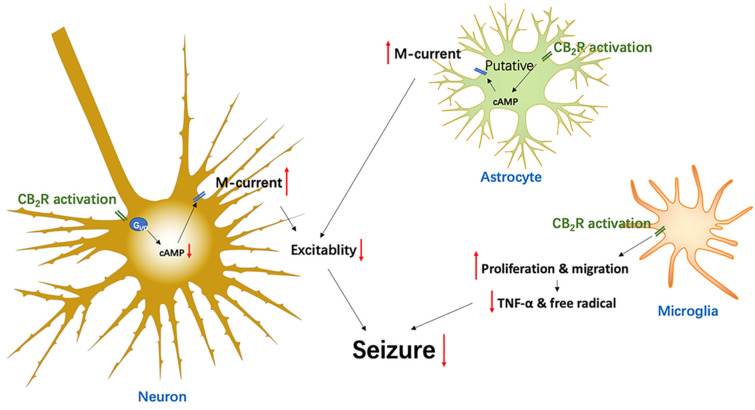
Diagram epitomizing the CB_2_R-associated mechanisms in the modulation of epileptic seizures.

In early studies, CB_2_Rs were found in the peripheral region, while CB_1_Rs were mainly expressed in the CNS, which led to a question of the existence of the CB_2_Rs in the CNS. Nowadays, with the development of more sensitive detection technologies, CB_2_Rs have been found in multiple brain regions in the CNS, though at a low level of expression compared to CB_1_Rs. However, CB_2_R expression and function are rapidly and profoundly increased under pathological conditions in the CNS. This attractive feature means that CB_2_R is considered as a disease-associated target, suggesting that it will greatly reduce the occurrence of CB_1_R’s side-effects through modulating the activity of CB_2_Rs to improve the neurological disorders. Drug-resistant epilepsy seriously affects the quality of life of patients, which highlights the need to invent more effective treatments. Although the underlying mechanisms of drug-resistant epilepsy are still unclear, several novel medicines to improve drug-resistant epilepsy have been developed. One example is the CB_1_ agonist, THC, which has been replaced by CBD due to its psychoactive side-effect. Epidiolex has been recently approved for the treatment of rare, severe forms of epilepsy by the USA’s Food and Drug Administration (FDA) [109]. Although this shows a promise of targeting the endocannabinoid system as a novel anti-seizure treatment, some harmful side-effects, including somnolence, diarrhea, appetite inhibition, and increased levels of hepatic transaminase [104] and blood pressure [105], limit its use. Considering that CB_2_Rs exhibit low expression levels in the brain under normal conditions, but are highly inducible during various disease states (including epilepsy), they appear to be an important substrate for neuroprotection [76]. Targeting CB_2_Rs will likely offer a novel therapeutic strategy for treating epileptic seizures without the typical CB_1_R-mediated side-effects [77]. Figure 1 summarizes the potential mechanisms of CB_2_R activation to inhibit seizures, that include reduced neuronal excitability by downregulating cAMP, and consequently enhanced M-currents in both neurons and astrocytes. In addition, CB_2_Rs can also regulate immune function and slow down neuroinflammatory responses. Together, it suggests that the CB_2_R may be an important target for controlling epileptic seizures.

## Figures and Tables

**Table 1:** Preclinical evidence for CB_2_R-mediated modulations in epilepsy.

Modulation Approaches	Seizure or Epilepsy Model	Response	References
-	PTZ induced seizures in heterozygous and homozygous CB2 knockout mice	Susceptibility	[84]
-	Handling-induced and spontaneous seizures	Susceptibility	[81]
2-Arachidonoylglycerol (2-AG)	Kainate induced seizures in CB_1_R and CB_2_R double-knockout mice	Suppressed excitatory	[12]
CB_2_ agonist beta caryophyllene	PTZ induced seizures in mice	Anticonvulsant activity	[85]
CB_2_ agonist beta caryophyllene	Induced by kainic acid (KA) seizure in mice	Decreased the seizure scores	[85]
CB_2_ antagonist AM630	Kainate induced seizures in CB1R knockout mice	Increased seizure susceptibility	[80]
CB_2_ agonist AM1241	PTZ induced seizures in rats	Increased seizure severity	[86]
CB_2_ agonist HU-308	DMCM and PTZ induced seizures in rats	No significant effect on seizure severity	[80]
CB2R agonist JWH133	PTZ induced seizures in mice	No significant effect on seizure severity	[84]
CB2R antagonist SR144528	PTZ induced seizures in mice	Increases seizure susceptibility	[84]
CB1/2R mixed agonist WIN 55,212-2	DMCM and PTZ induced seizures in rats	Anticonvulsant effects	[80]

## Data Availability

Not applicable.
